# Enrichment of Acid-Associated Microbiota in the Saliva of Type 2 Diabetes Mellitus Adults: A Systematic Review

**DOI:** 10.3390/pathogens12030404

**Published:** 2023-03-02

**Authors:** Jéssica Alves Cena, Letícia Gonçalves Reis, Ana Karolina Almeida de Lima, Camilla Pedrosa Vieira Lima, Cristine Miron Stefani, Naile Dame-Teixeira

**Affiliations:** 1 Department of Dentistry, School of Health Sciences, University of Brasília, Federal District, Brasília 70910-900, Brazil; 2 Division of Oral Biology, School of Dentistry, University of Leeds, Leeds LS9 7TF, UK

**Keywords:** oral microbiome, diabetes mellitus, dental caries, microbiota, saliva

## Abstract

It could conceivably be hypothesized that a link exists between an altered microbiota due to local hyperglycemia and the increased risk of caries in diabetes mellitus (DM). This systematic review aimed to perform a cross-study comparison into the salivary microbiota of adults with type 2 diabetes mellitus (T2D) compared to adults without T2D, particularly focusing on the abundance of acid-associated bacteria. This report follows PRISMA (Preferred Reporting Items for Systematic Reviews and Meta-Analyses). Studies using next-generation sequencing and other molecular techniques are included. The methodological quality of individual studies was assessed using appropriate Joanna Briggs Institute tools. The certainty of the evidence considering the effect direction was evaluated using the GRADE approach. From 2060 titles retrieved, 12 were included in the data synthesis, totalling 873 individuals with T2D and controls evaluated across the literature. Weighted averages of blood glucose levels (HbA1c—fasting blood glucose) were 8.21%—172.14 mg/dL and 5.12%—84.53 mg/dL for T2D and controls, respectively. In most studies, the relative abundance of acidogenic and aciduric bacteria was higher in diabetics when compared to their normoglycaemic controls. Whilst the evidence certainty was very low, there was a consistent *Proteobacteria* depletion and *Firmicutes* enrichment in T2D. As for the acid-associated genera, there was consistent enrichment of *Lactobacillus* and *Veillonela* for T2D. *Tannerella*/*T. forsythia* was enriched in T2D saliva, but the certainty is low. Further well-designed cohorts are needed to clarify the distribution of acid-associated microorganisms in the saliva of adults with T2D and how this can be clinically manifested (PROSPERO = CRD42021264350).

## 1. Introduction

According to the International Diabetes Federation’s Atlas, diabetes mellitus (DM) affects 537 million people worldwide. It is a growing condition, with an expected incidence of 643 million people by 2030 and 783 million by 2045 [1]. More than 90% of people diagnosed with DM suffer from type 2 DM (T2D) [2,3,4], which is associated with tissue damage, dysfunction of several body systems, and an increased risk of oral diseases [5,6]. Salivary fluid reflects altered serum glucose levels and inflammatory cytokines, both of which are related to oral manifestations of DM [7,8,9,10]. Glucose transporters are found in salivary glands, which means that glucose is transported from the blood to saliva [11]. In addition, the salivary flow, which is critical to maintaining oral health, is reduced in people living with DM [12]. 

There is an association between dental caries and DM. It was shown that T2D may be a risk factor for increased number of teeth affected by coronal and root caries in adults [13]. This can be influenced by reduced salivary flow and altered microbiota due to local hyperglycaemia [14]. The increased availability of glucose in saliva creates an environment that can favour the growth of caries-associated acidogenic and aciduric microorganisms [3,15]. This hypothesis has been tested in adolescents, where high salivary glucose levels were associated with an increase in acidogenic microorganisms in diabetic saliva and an increased risk of caries [11]. In this study, more than 50% of the bacteria tested using the checkerboard DNA–DNA hybridization approach were *Neisseria mucosa*, *Eikenella corrodens*, *Streptococcus mitis*, *Prevotella melaninogenica*, *Veillonella parvula*, *Streptococcus oralis*, and *Streptococcus salivarius*, with several acidogenic microorganisms enriched in hyperglycemic saliva [11]. Nevertheless, there is still a lack of evidence on adults with T2D, and the generalizability of age on this issue may be problematic.

A comprehensive understanding of how DM contributes to changes in the salivary microbial profile is still lacking [16,17]. Because saliva can reflect the entire oral micro-ecosystem, understanding the composition of the saliva microbiota would be more useful in understanding individuals’ general health prospects than specific site biofilms [18,19]. Hence, this systematic review aimed to compare the salivary microbiota of adults with type 2 diabetes mellitus (T2D) to that of adults without T2D, with a particular focus on the abundance of acid-associated bacteria. The hypothesis is that there may be an enrichment of acidogenic and aciduric microorganisms in the saliva of T2D individuals due to increased salivary glucose levels, which could be involved in oral manifestations of T2D such as caries, periodontal disease, and fungal infections.

## 2. Materials & Methods 

### 2.1. Study Design

The acronym PECOs (Population, Exposure, Comparison, Outcomes, and Studies) [20] was used to build the following research question: “Is there an enrichment of acid-associated (aciduric and/or acidogenic microorganisms) in the saliva of adults with T2D when compared to individuals without T2D or with controlled T2D?”, where P = saliva of adults, E = T2D or uncontrolled T2D, C = individuals without T2D or controlled T2D, O = salivary microbiota, the abundance of aciduric, and/or acidogenic microorganisms, and S = clinical/observational studies.

### 2.2. Eligibility Criteria and Search Strategy

Studies must assess the salivary microbiome in individuals with T2D and have a control group of individuals without DM (or controlled T2D) to be eligible. The relative abundance of microorganisms should be evaluated using molecular microbiology techniques, such as species-specific PCR, DNA–DNA hybridization, and nucleic acid sequencing. Exclusion criteria were (1) non-human samples; (2) samples other than saliva (e.g., biofilm, crevicular fluid); (3) samples from children or adolescents; (4) samples including individuals with type 1 or gestational DM; (5) studies not using molecular methods for analysis of the salivary microbiome (e.g., culture methods); and (6) studies involving animals, in vitro studies, reviews, book chapters, opinions, letters, conference abstracts, study protocols, case reports, and case series.

A detailed search strategy was developed and adapted for each of the following bibliographic databases: PubMed/MEDLINE, EMBASE, LILACS, Web of Science, Scopus, and grey literature (Google Scholar, Livivo, and ProQuest Dissertations & Theses Global) (Supplementary Table S1). Additional expert searches and manual searches of reference lists were performed. Duplicate studies were removed using the reference manager software (EndNote^®^, Thomson Reuters, Philadelphia, NC, USA).

### 2.3. Studies Selection

Two independent reviewers (L.G.R. and J.A.C.) selected the studies in two phases using the Rayyan data manager (Rayyan QCRI^®^; Qatar Computer Research Institute, Doha, Qatar): (1) selection based on titles and abstracts and (2) reading of the full texts. Any discrepancies were resolved through consensus. 

### 2.4. Data Collection and Analysis

Two reviewers (L.G.R. and J.A.C.) extracted information from the selected articles, which was then revised for accuracy by a third reviewer (N.D-T.). The data recorded were: year and country of the study, number of participants, caries and periodontal disease status, details on the saliva collection and its clinical diagnosis (including hyposalivation and xerostomia), DNA extraction method, primers and the molecular approach employed, the database used for mapping genetic sequences (when applicable), taxonomy at the phylum level, taxa related to low pH, the relative abundance of acid-associated microorganisms for each group of participants, diabetes diagnosis, and glucose levels in all participants (blood and saliva).

Weighted averages of HbA1c and fasting blood glucose (FBG) levels were calculated for both the T2D and control groups across all included studies. The overall microbiota and the acid-associated microbiota were evaluated based on their effect direction (whether they were significantly enriched or depleted in T2D) according to the following categories: phylum, acid-lactic bacteria (LAB), main saccharolytics that produce short-chain fatty acids (such as butyrate, succinate, acetate, and propionate), and acid-associated microorganisms (such as lactate metabolizers).

### 2.5. Methodological Quality Assessment

Two reviewers (L.G.R. and J.A.C.) independently evaluated the methodological quality of the studies using the Joanna Briggs Institute (JBI) checklist for cross-sectional studies [21]. The checklist consisted of eight items, from which items 1–4 were considered critical for this systematic review. Items 5–8 were considered non-critical outcome-related criteria. Low methodological quality was indicated by at least one “no” and one “not clear” or two “not clear” in critical items, or two “not clear” and one or more “no” in non-critical items. Before applying the instrument, the research team discussed the critical and non-critical items, as well as the classification system, as described in the JBI Reviewer’s Manual [21]. Disagreements were resolved by consensus.

### 2.6. Certainty of the Evidence

The analysis of the risk of bias, inconsistency, imprecision, indirectness, and publication bias was performed for reaching the certainty of evidence through the GRADE (Grading of Recommendations, Assessment, Development, and Evaluation) approach for narrative synthesis [22]. Only taxa with effect direction were analysed by the GRADE approach.

## 3. Results 

### 3.1. Characteristics of Selected Studies

**Figure 1:**
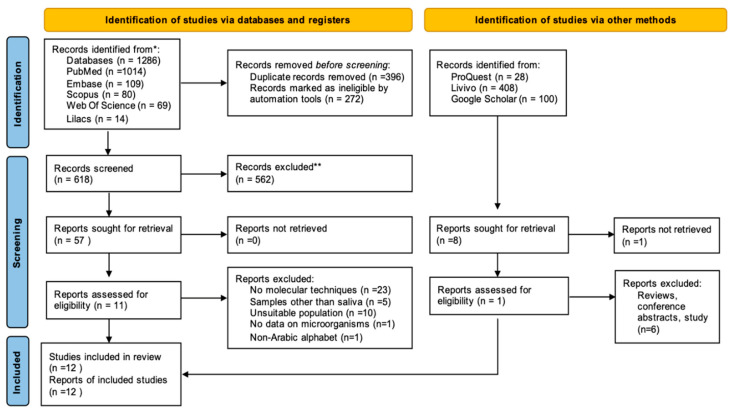
Flowchart of the study selection according to the PRISMA (Preferred Reporting Items for Systematic Reviews and Meta-Analyses) guidelines.

After the initial screening of titles and abstracts, 59 were selected for full-text reading. Among them, 12 studies were eligible for the qualitative synthesis. These studies were conducted in 10 different countries, including Brazil (1), Portugal (1), United Arab Emirates (1), India (1), Thailand (1), Pakistan (1), Japan (1), the United States of America (1), Saudi Arabia (1), and China (3). The process of study identification, inclusion, and exclusion is presented in Figure 1. Detailed information on the reasons for exclusion after full-text reading is provided in Supplementary Table S2.

A total of 418 individuals with T2D and 494 individuals without DM were included in the analysis. Sample sizes ranged from 3 to 79 individuals with T2D and 10 to 80 controls. The classification of individuals as cases or controls followed the criteria of each included study separately. Table 1 shows the results of the methodological quality assessment of the individual studies, the type and method of sample collection, and the microbial analysis approach. Six articles were classified as having low methodological quality due to a lack of detailed descriptions of participants and study scenario, as well as a lack of identification and strategies for dealing with confounding factors. Three studies were classified as high quality, and three as moderate quality (see Supplementary Table S3 for details).

The level of HbA1c was the most commonly used criterion for DM diagnosis, although the cut-off points were varied (>6%, 6.5%, 7%, or 8%). We calculated the weighted average of HbA1c and FBG levels across all included studies to confirm the global difference between groups. For participants with T2D, these levels were 8.21% and 172.14 mg/dL (9.56 mmol/L), respectively. The average levels of HbA1c and FBG for participants without T2D were 5.12% and 84.53 mg/dL (4.7 mmol/L), respectively. Some studies used the participant’s self-reported diagnosis of DM to define groups [2,23], which can generate bias if DM is not confirmed through clinical and laboratorial exams, as recommended by the IDF, ADA, or WHO. In one of the studies [2], the validation of DM diagnosis by blood glucose tests was only performed in individuals who self-reported a history of DM, meaning that control individuals could also have high levels of blood glucose. A single study declared that they followed the ADA criteria to classify a participant as “controlled T2D” (“*diabetes in remission is a return of HbA1C to less than 6.5% that occurs spontaneously or following an intervention, which persists for at least three months*”). The small sample size is another issue that may bias the data of some studies [24,25].

Most studies reported the participants’ periodontal status [2,14,23,24,26,27,28]. One study reported the DMFT (decayed, missing, and filled teeth) for three participants with T2D [25], while other studies reported the mean DMFT in individuals with or without T2D [14,29]. Only one study reported glucose concentrations in saliva [14]. Unstimulated saliva was the most common method of sample collection. A single study addressed data on the salivary flow diagnosis (normal flow, hyposalivation, asialia) [14].

### 3.2. Salivary Microbiota 

The partial amplification and sequencing of the 16S rRNA gene was the most commonly used approach. The sequenced regions of the 16S rRNA gene ranged from V2 to V9, with V3 and V4 being the most commonly used. Four studies used qPCR (real-time PCR) analysis, with or without a sequencing approach [23,24,27,28]. Regarding the DNA extraction, two studies employed manual protocols [4,25], two used automated protocols [2,27], and eight used commercially available extraction kits [14,15,23,24,26,28,29,30] (Table 1).

A consensus regarding differences in alpha and beta diversity between the DM and control groups was not reached. One study reported a remarkable increase in alpha diversity within the DM group when compared to normoglycemic controls [24], whereas others observed the opposite trend [2,14,27] or found no significant differences [4,25,28,29].

Table 2 displays the directional effect of the salivary microbiota at various taxonomic levels. To enable cross-study comparison, microorganisms were analysed based on the following categories: phyla in general, lactic acid bacteria (LAB), main saccharolytic bacteria producing butyrate or other short-chain fatty acids (succinate, acetate, propionate), and acid-associated microorganisms (lactate metabolizers, other acidurics). Variations in the diversity and abundance of the acidogenic/aciduric microbiota were observed among studies and groups. The same bacteria comprising the core of the salivary microbiome were found in both groups, with no pronounced changes in abundance. At the phyla level, *Firmicutes*, *Bacteroidetes*, and *Proteobacteria* were the most predominant for both, diabetics and controls. *Streptococcus*, *Veillonella*, and *Fusobacterium* were among the most common genera. Interestingly, when a significant difference between groups was observed, some well-known acidogenic taxa were overrepresented in T2D, such as *Bifidobacteriaceae*, *Bifidobacterium*, *Scardovia*, *Parascardovia*, *and Lactobacillus* (Table 2). 

The certainty of the evidence presented in the outcome (main taxa that displayed an effect direction) was evaluated using the GRADE approach, as shown in Table 3. Based on the GRADE recommendation, the certainty of observational studies began at a low level, and no upgrades were applicable in the context of this review. The direction and degree of the effect varied across studies. In general, the findings indicated a slight increase in the targeted microorganisms in individuals with DM, or no significant difference between groups.

Nonetheless, the relative abundance of certain acid-associated bacteria may be higher in individuals with T2D compared to those without T2D. *Lactobacillus* (acidogenic) and *Veillonela* (lactate-metabolizing, aciduric) consistently displayed an effect direction towards T2D, albeit the evidence was very uncertain [15,25,31]. Additionally, *Prevotella* and *Fusobacterium* were enriched in the saliva of adults with T2D compared to those without T2D or with controlled T2D, but the evidence was very uncertain. At the phylum level, there was a clear and consistent depletion of *Proteobacteria* (Gram-negative, often associated with proteolytic metabolism) and enrichment of *Firmicutes* (including lactic-acid bacteria) in T2D (very low certainty). Meanwhile, *Tannerella*/*T. forsythia* was enriched in T2D (low certainty).

## 4. Discussion 

In a previous study, we demonstrated a significant correlation between blood and salivary glucose levels, as well as their impact on increasing the number of caries lesions in adults with T2D [10]. This led us to question whether an acid-associated microbiota could be enriched in saliva due to high levels of salivary glucose. We conducted a systematic review of the literature to respond to this question. The weighted average of HbA1c and FBG levels across all studies included in this review confirmed the presence of hyperglycemia in the test groups, which was associated with the enrichment or depletion of some bacterial taxa. This included a significant enrichment of certain acid-associated bacteria, thus supporting our initial hypothesis.

Characterizing the salivary microbiota of T2D is a challenge [9,31,32]. Apart from the expected individual variations, external factors such as diet and geographical differences can influence oral microbiome composition [2,14,23,24,26,27,33]. Coexisting conditions, such as periodontal disease, caries, obesity, hyposalivation, polypharmacy, and other comorbidities can introduce biases in studies evaluating the salivary microbiota. For instance, periodontal disease impacts the oral microbiome and the T2D levels [34]. Despite this, only half of the included studies in this review reported on individuals’ periodontal status [2,14,23,24,26,27]. Our study confirmed a significant enrichment of the periodontal disease-associated bacteria *Tannerella* in T2D saliva. 

Saliva is not an optimal sample for identifying differences between the periodontal microbiome of diabetic and non-diabetic individuals. Nevertheless, the salivary fluid can still be useful in exploring the influence of other clinical parameters in the oral microbiome, such as fluctuations in pH, glucose levels, and flow volumes. As the salivary microbiome reflects the entire oral environment, it can be more representative for investigating shifts from homeostasis to dysbiosis related to systemic conditions. In addition, saliva may be better for detecting the enrichment of acid-associated microorganisms, and this could shed light on the association between dental caries and T2D. These findings might be consistent with other studies linking an increased risk for candidiasis in diabetic patients and their oral mycobiome, although we could not evaluate the acidogenic mycobiome here, as most of the included studies used prokaryote analysis of 16S rRNA.

Most studies did not take into account confounding factors, such as salivary hypofunction and the individual’s dietary habits. There is a consensus that the combination of DM, poor glycemic control, and polypharmacy are associated with xerostomia and the hypofunction of salivary glands in T2D patients [12,35,36]. Consequently, changes in the main oral microorganisms, as detected here, can be observed between individuals with xerostomia and those with normal salivary function [37]. In terms of dietary habits, sugar intake may contribute to an increase in blood glucose levels [13] and a decrease in oral pH, promoting the growth of acid-associated bacteria [38]. These facts highlight the need for a global study of the microbiota in populations with distinct dietary habits for a proper assessment of their importance in the human microbiome architecture [39]. 

At the phylum level, three studies did not find significant differences in the abundance of *Firmicutes* between T2D and control groups. Several factors could explain the discrepant result, such as the presence of individuals who had taken antibiotics within one month before the study in the no-T2D group [27], the categorization of the no-T2D group based on their weight [27], the method of saliva collection, and the type of molecular method used for microbial identification. For example, one of these studies collected stimulated saliva after a tongue smear [26]. In addition, the variation in the type of primers and 16S rRNA regions used could have influenced the results [40,41]. On the other hand, *Proteobacteria* showed a significant effect direction, and the single divergent study used a different molecular approach (RT-PCR), while all the others were based on 16S rRNA sequencing.

Surprisingly, only one study reported salivary glucose levels, which were evaluated using stimulated saliva and a colorimetric kit. The levels were categorized as high (≥0.35 mg/dL) or low (<0.35 mg/dL), and the average salivary glucose concentration was higher in the T2D group (0.84 mg/dL) than in the control group (0.37 mg/dL). Goodson et al. (2017), comparing adolescents with high and low salivary glucose levels, found that higher salivary glucose levels were associated with lower bacterial counts, particularly of non-saccharolytic bacteria, such as *Prevotella* spp. Although with very uncertain evidence, our systematic review showed a divergent result for this taxon in adults.

Several limitations may introduce bias into this cross-study analysis, including the lack of a clear definition of DM diagnosis in the included studies. In some studies, individuals classified as diabetics used drugs such as metformin and insulin, and they were not always categorized as controlled or uncontrolled T2D. This could result in misinterpretation of the data from the salivary microbiota. However, due to the weighted averages of HbA1c and FBG, we believe that this issue affected the results at the minimum level. Additionally, most of the included studies were cross-sectional, making it impossible to determine how long it takes for the microbiota to change. Cohort studies would be valuable in understanding the transition from homeostatic to dysbiotic state in the diabetic salivary microbiome. Furthermore, a quantitative analysis (meta-analysis) was not feasible due to the lack of standardization across reports on microbiological data [42], emphasizing the importance of developing a specific guideline.

## 5. Conclusions

Studies with molecular microbiology analyses suggest that some typically acidogenic and aciduric microbiota may be enriched in the salivary microbiome of individuals with T2D when compared to controls without T2D, although the evidence is uncertain. The effect direction consistently showed enrichment of *Firmicutes*, *Lactobacillus*, *Veillonela*, and *Tannerella*/*T. forsythia* in T2D, and a depletion of *Proteobacteria*. However, it was not possible to associate these results with increased salivary glucose levels due to the lack of information within the included studies. Future well-designed cohorts are needed to clarify the distribution of acid-associated microorganisms in the saliva of adults with T2D, and how this can be clinically manifested.

## 6. Records

This systematic review was reported following the *Preferred Reporting Items for Systematic Reviews and Meta-Analyses* (PRISMA) [43] and its extension *Synthesis Without Meta-analysis* (SWiM) [44]. The review protocol was registered in the International Prospective Register of Systematic Reviews (PROSPERO) under number CRD42021264350.

## Figures and Tables

**Table 1:** Characteristics of the included studies (n = 12 studies) evaluating the salivary microbiota of individuals with and without type 2 diabetes mellitus (T2D).

Study	Mq *	Group with DM	Controls	Salivary Collection	Microbiota Assessment
Almeida-Santos, 2021 Portugal	−	T2D reported and treated for more than 14 years (HbA1c = 7.2% ± 1.0) n = 25, mean age: 62.7 ± 7.0 yo.	Those reporting no T2D n = 25, mean age: 60.0 ± 8.8 yo.	Not reported **	16S rRNA V3–V4 region
Al-Rawi, 2017 United Arab Emirates	−	Obese with T2D (FBG = 205.5 ± 83.9 mg/dL) n = 26, mean age: 51.1 ± 5.7 years Obese without T2D (FBG = 106.2 ± 24.9 mg/dL) n = 26, mean age: 47.9 ± 5.7 yo.	Non-obese and non-diabetic (FBG = 94 ± 17.1 mg/dL) n = 26, mean age: 47.4 ± 5 yo.	Whole saliva, unstimulated	RT-PCR using taxon-specific primers targeting six proteolytic bacteria, and including a universal 16S rRNA primer
Anbalagan, 2017 India	−	T2D reported (HbA1c = 11.33% ± 1.56) n = 24, mean age: 50.5 ± 14.72 yo.	Those reporting no T2D (HbA1C < 6.5%) n = 10	Whole saliva, unstimulated	16S rRNA V6 region
Chumponsuk, 2021 Thailand	+	T2D according to FBG (FBG= 119.37 ± 37.12 mg/dL) n = 25, mean age: 50.64 ± 13.75 yo.	Non-diabetics were categorized into 3 groups according to BMI (FBG = 96.11 ± 6.7 mg/dL) n = 80, mean age: 44.82 ± 2.5 yo.	Whole saliva, unstimulated	RT-PCR using several taxon-specific primers targeting 16S rRNA
Kori, 2020 Pakistan	−	T2D reported (HbA1c = 8.3% ± 1.7) n = 49, mean age: 53.1± 7.9 yo.	Those reporting no T2D (HbA1c= 5.2% ± 0.4) n = 50, mean age: 39.7 ± 11.8 yo.	Whole saliva, unstimulated	16S rRNA V3-V4 region
Vieira Lima, 2022 Brazil	**++**	T2D reported and treated (HbA1c = 8.83% ± 2.02, FBG= 148.13 ± 65.43 mg/dL) n = 23, mean age: 58.52 ± 8.5 yo.	Those reporting no T2D (HbA1c = 5.25% ± 0.41, FBG = 89.67 ± 13.07 mg/dL) n = 25, mean age: 43.13 ± 12.98 yo.	Whole saliva, unstimulated and stimulated	16S rRNA V4 region
Liu, 2021 China	+	Reported and untreated T2D (HbA1c = 8.51% ± 2.33) n = 24, mean age: 47(33–65) yo.	Those reporting no T2D (HbA1c = 5.25% ± 0.41) n = 21, mean age: 47.24(35∼61) yo.	Whole saliva, unstimulated	16S rRNA V3-V4 region
Ogawa, 2017 Japan	−	Those who reported treatment for T2D (HbA1c = 8.51% ± 2.33) n = 3, mean age: 85.3 ± 4.5 yo.	Those reporting no T2D n = 12, mean age: 83.9 ± 8.4 yo.	Unstimulated saliva	16S rRNA V4 region
Sabharwal, 2018 United States	−	Self-reported T2D (HbA1c = 7.37% ± 0.30) n = 79, mean age: 52.9 ± 2.13 yo.	Those reporting no T2D n = 64, mean age: 39.3 ± 8.5 yo.	Unstimulated saliva	16S rRNA V3-V4 region
Saeb,2019 Saudi Arabia	**++**	T2D reported and treated (HbA1c = 9.5% ± 0.83) for more than 11 years n = 15, mean age: 51.2 ± 3.14 yo.	Those reporting no T2D n = 19, mean age: 41–56 yo.	Whole saliva spat out after tongue rubbing (OM501 Kit for Microbial DNA Analysis)	16S rRNA qPCR V2-4 and V6-9 regions
Sun, 2020 China	**++**	Reported and treated T2D (HbA1c = 7.7% ± 2.06) n = 75, mean age: 54.96 ± 8.95 yo.	Those with no reported T2D (HbA1c = 4.96% ± 0.15) n = 58, mean age: 36.66 ± 11.15 yo.	Unstimulated saliva, after rinsing with tap water	16S rRNA qPCR V3-V4 region
Yang, 2020 China	+	T2D reported or reported and treated (FBG = 11.58 ± 0.99 mmol/L) n = 70, mean age: 54.62 ± 12.13 yo.	Those with no reported T2D (FBG = 5.38 ± 0.48 mmol/L) n = 32, mean age: 49.18 ± 8.72 yo.	Unstimulated saliva, after rinsing with sterile distilled water	16S rRNA V3-V4 region

Mq * = Methodological quality, where **++** low risk of bias; + moderate risk of bias; − high risk of bias (according to JBI criteria); ** Not reported = authors did not describe the collection method; “16S rRNA” means amplification and partial sequencing of the 16S rRNA gene. DM = Diabetes mellitus; T2D = Type 2 diabetes mellitus; FBG = Fasting Blood Glucose; HbA1c = Glycated haemoglobin; yo. = years-old.

**Table 2:** Analysis of the general salivary microbiota and that typically acidogenic or acid-related in individuals with and without type 2 diabetes mellitus (T2D).

Microorganism **	Al-Rawi l, 2017	Sun, 2020	Anbalagan, 2017	Yang, 2020	Kori, 2020	Chumponsuk,2021	Almeida-Santos,2021	Liu, 2021	Ogawa, 2017	Sabharwal, 2019	Saeb, 2019	Vieira Lima, 2022
**Phyla in general**												
*Firmicutes*				↑	↑	NS		NS		↑ *	NS	
*Bacterioidetes*		↑ *		↑	NS			NS	↓ *		NS	
*Actinobacteria*					NS	NS	NS			↑ *	NS	↓ *
*Proteobacteria*				↓	↓	NS		NS		↓ *		↓
*Spirochaetota*					NS		NS					
*Euryarchaeota*												
*Fusobacteria*				↑	NS			NS				
**Lactic acid bacteria (LAB)**												
*Streptococcus*		↓ *	↑ *			NS					NS	NS
*Lactobacillus* spp.		↑ *		↑		NS					NS	NS
*Bifidobacteriaceae*, *Bifidobacterium*						NS		↑ *			NS	NS
*Scardovia*, *Parascardovia*								↑ *				NS
*Enterococcus*												
Other LAB (*Lactococcus*, *Leuconostoc*, *Pediococcus*, *Vagococcus*, *Aerococcus*, *Tetragenecoccus*, *Carnobacterium*)												
**Bacteria producing butyrate or other short chain fatty acids (succinate, acetate, propionate)**												
*Butyvibrio*												↓
*Bacteroides*						NS					NS	
*Prevotella*		↑ *		↑	↑	NS		↓ *			NS	
*Alloprevotella*									↓ *			
*Paraprevotella*												
*Fusobacterium*	↑		↑ *			NS			↑ *			
*Tannerella*, *T. forsythia*	↑	↑ *										↑
*Treponema*, *T. denticola*	NS							↓ *				
**Acid-associated microorganisms (lactate metabolizers, other acidurics)**												
*Veillonella*			↑ *		↑	NS			↑ *		NS	NS

↑ Significant enrichment in T2D when compared to healthy controls; ↓ Significant depletion in T2D when compared to healthy controls; NS = non-significant differences reported by the included studies; * Reports taxon increase in one of the groups, but without explicit statistical analysis; ** Blank boxes mean that the studies did not report increased or decreased presence of the taxon. Some taxa were screened but no studies reported them.

**Table 3:** Certainty of the evidence assessed by the GRADE system for the phyla in general, as well as for the acid-associated bacteria. Only taxa with some effect directions were included.

Certainty Assessment							Impact	Certainty
№ of Studies	Study Design	Risk of Bias	Inconsistency	Indirectness	Imprecision	Other Considerations		
***Firmicutes***								
12	Observational	Not serious ^a^	Very serious ^b^	Not serious	Serious	None	The evidence is very uncertain about the enrichment of *Firmicutes* in the saliva of adults with T2D when compared to individuals without T2D or with controlled T2D.	⨁◯◯◯ Very low
***Proteobacteria***								
12	Observational	Not serious ^a^	Very serious ^b^	Not serious	Serious ^c^	None	The evidence is very uncertain about the depletion of *Proteobacteria* in the saliva of adults with T2D when compared to individuals without T2D or with controlled T2D.	⨁◯◯◯ Very low
***Prevotella***								
12	Observational	Not serious ^a^	Very serious ^b^	Not serious	Serious ^c^	None	The evidence is very uncertain about the enrichment of *Prevotella* in the saliva of adults with T2D when compared to individuals without T2D or with controlled T2D.	⨁◯◯◯ Very low
***Fusobacterium***								
12	Observational studies	Not serious ^a^	Very serious ^b^	Not serious	Serious ^c^	None	The evidence is very uncertain about the enrichment of *Fusobacterium* in the saliva of adults with T2D when compared to individuals without T2D or with controlled T2D.	⨁◯◯◯ Very low
***Veillonella***								
12	Observational studies	Not serious ^a^	Very serious ^b^	Not serious	Serious ^c^	None	The evidence is very uncertain about the enrichment of *Veillonella* in the saliva of adults with T2D when compared to individuals without T2D or with controlled T2D.	⨁◯◯◯ Very low
***Lactobacillus* spp.**								
12	Observational	Not serious ^a^	Very serious ^b^	Not serious	Serious ^c^	None	The evidence is very uncertain about the enrichment of *Lactobacillus* spp. in the saliva of adults with T2D when compared to individuals without T2D or with controlled T2D.	⨁◯◯◯ Very low
***Tannerella*/*T. forsythia***								
12	Observational	Not serious ^a^	Serious ^b^	Not serious	Serious ^c^	None	*Tannerella*/*T. forsythia* may be enriched in the saliva of adults with T2D when compared to individuals without T2D or with controlled T2D, although the certainty is low.	◯⨁◯◯ Low

(^a^) There was a comprehensive search in databases, grey literature, and manual search, attempting to avoid publication bias; However, some included studies had low and moderate methodological quality. (^b^) The direction and magnitude of the effect varied between different studies. (^c^) The optimal information size (OIS) is borderline and there were small effects or ‘no effects’ reported in studies. ⨁◯◯◯—very low certainty of evidence; ◯⨁◯◯—low certainty of evidence; ◯◯⨁◯—moderate certainty of evidence; ◯◯◯⨁—high certainty of evidence.

## Data Availability

Data is contained within the article or supplementary material.

## Supplementary Materials

The following supporting information can be downloaded at: https://www.mdpi.com/article/10.3390/pathogens12030404/s1, Table S1: Complete search strategies for each database and grey literature; Table S2: Excluded studies and reason for exclusion (n = 47); Table S3: Quality assessment of the individual included studies (n = 12) using The Joanna Briggs Institute Critical Appraisal Checklist for Cross-sectional Studies. References [3,5,8,11,17,45,46,47,48,49,50,51,52,53,54,55,56,57,58,59,60,61,62,63,64,65,66,67,68,69,70,71,72,73,74,75,76,77,78,79,80,81,82,83,84,85] are cited in the supplementary materials.
