# Supplementary material for: Enrichment of Acid-Associated Microbiota in the Saliva of Type 2 Diabetes Mellitus Adults: A Systematic Review
Source: Pathogens. 2023 Mar 2;12(3):404. doi: 10.3390/pathogens12030404 (PMC10051551; doi:10.3390/pathogens12030404)
Supplement: Supplementary file 1 [file pathogens-12-00404-s001.zip › pathogens-2235223-supplementary.pdf]

Enrichment of acid-associated microbiota in the saliva of type 2 diabetes mellitus adults: A systematic review

Supplementary material

Supplementary Table S1. Complete search strategies for each database and grey literature.

Database	Search strategy<br>(search date: January 19th, 2023)	Results
**MEDLINE via PubMed**	(("Microbiota" OR ("Microbiota"[Mesh]) OR "Community, Microbial" OR "Microbial Community Compositions" OR "Community Structure, Microbial" OR "Microbiome, Human" OR "microbiota" OR "microbiotas" OR "Microbiotas" OR "Microbiome" OR "microbiome" OR "microbiomes" OR "Microbiomes" OR "Microbiology" OR "Microbial Community" OR "Microbial community" OR "microbial community" OR "Microbial Communities" OR "Microbial communities" OR "microbial communities" OR "Human microbiome" OR "human microbiome" OR "Human Microbiome" OR "DNA, Bacterial" OR "DNA Bacterial" OR "Salivary microbiota" OR "salivary microbiota" OR "salivary microbiome" OR "16S" OR "16S rRNA" OR ("RNA, Ribosomal, 16S"[Mesh]) OR "rRNA, 16S" OR "16S Ribosomal RNA" OR "RNA, 16S Ribosomal" OR "Ribosomal RNA, 16S" OR "16S rRNA gene" OR "16S rRNA sequencing" OR "Biodiversity" OR "biodiversity" OR "biodiversities" OR "Biodiversities" OR "Metagenome" OR ("Metagenome"[Mesh]) OR "Metagenomes" OR "metagenomes" OR "metagenome" OR "Metagenomics" OR "metagenomics" OR "High-Throughput Nucleotide Sequencing" OR "High Throughput Nucleotide Sequencing" OR "Next-generation sequencing" OR "next-generation sequencing" OR "next generation sequencing" OR "Next generation sequencing" OR "Oral microbiota" OR "Oral Microbiota" OR "oral microbiota" OR "microbial interactions" OR "Microbial interactions" OR "Microbial Interactions" OR "host-microbial interactions" OR "host microbial interactions" OR "Saliva / microbiology" OR "aciduric microbiota" OR "Aciduric microbiota" OR "metabolomics" OR "metabolomic" OR "Host microbiota" OR "host microbiota" OR "host microbiome" OR "Host microbiome") AND ("Type 2 Diabetes Mellitus" OR ("Diabetes Mellitus, Type 2"[Mesh]) OR "Diabetes Mellitus, Noninsulin-Dependent" OR "Diabetes Mellitus, Non Insulin Dependent" OR "Diabetes Mellitus, Non-Insulin-Dependent" OR "Diabetes Mellitus, Type II" OR "NIDDM" OR "Diabetes Mellitus, Noninsulin Dependent" OR "Type 2 Diabetes Mellitus" OR "salivary glucose" OR "Type 2 Diabetes" OR "Diabetes, Type 2" OR "Diabetes Mellitus , Type 2" OR "Diabetes Mellitus Type 2" OR "Diabetes Mellitus type 2" OR "Diabetes Mellitus, type 2" OR "T2DM" OR "Diabetes Mellitus" OR "Diabetes mellitus" OR ("Diabetes Mellitus"[Mesh]) OR "diabetes mellitus" OR "diabetes" OR "Diabetes" OR "Hyperglycemia" OR "Hyperglycemic")) AND ("Adults" OR ("Adult"[Mesh]) OR "adults" OR "adult" OR "Adult" AND "humans" OR ("Humans"[Mesh]) AND "Saliva" OR ("Saliva"[Mesh]) OR "Salivas" OR "salivas" OR "Salivary" OR "salivary" OR "Salivary samples" OR "salivary samples" OR "saliva sample" OR "Saliva sample" OR "saliva samples" OR "Saliva samples" OR "Microbial markers" OR "microbial markers" OR "Periodontal Pocket" OR ("Periodontal Pocket"[Mesh]) OR "Pocket, Periodontal" OR "Periodontal Pockets" OR "Pockets, Periodontal" OR	1014

	"periodontal pocket" OR "Periodontitis" OR ("Periodontitis"[Mesh]) OR "periodontitis" OR "Periodontal disease" OR ("Periodontal Diseases"[Mesh]) OR "Disease, Periodontal" OR "Diseases, Periodontal" OR "periodontal disease" OR "Chronic periodontitis" OR "Aggressive periodontitis" OR "Subgingival" OR "Dental caries" OR ("Dental Caries"[Mesh]) OR "Dental Decay" OR "Decay, Dental" OR "Carious Lesions" OR "Carious Lesion" OR "Lesions, Carious" OR "Caries, Dental" OR "Carious Dentin" OR "Dentin, Carious" OR "Dental White Spot" OR "White Spot, Dental" OR "Dental White Spots" OR "dental caries" OR "Root caries" OR "root caries" OR ("Root Caries"[Mesh]) OR "Caries, Root" OR "Caries, Cervical" OR "Cervical Caries" OR "Caries" OR "caries")	
**Web of Science**	("Adults" OR "Adult") AND ("Saliva" OR "Salivary" OR "Salivary samples" OR "Saliva sample" OR "Periodontal Pocket" OR "Periodontitis" OR "Periodontal Diseases" OR "Disease, Periodontal" OR "periodontal disease" OR "Chronic periodontitis" OR "Aggressive periodontitis" OR "Subgingival" OR "Dental caries" OR "Carious Lesions" OR "Root caries" OR "Cervical Caries" OR "Caries") AND ("salivary glucose" OR "Diabetes Type 2" OR "Diabetes Mellitus" OR "T2DM" OR "Diabetes" OR "Hyperglycemia" OR "Hyperglycemic") AND ("Microbiota" OR "Community, Microbial" OR "Microbial Community Compositions" OR "Community Structure, Microbial" OR "Microbiome, Human" OR "Microbiome" OR "Human microbiome" OR "DNA Bacterial" OR "Salivary microbiota" OR "salivary microbiome" OR "16S" OR "16S rRNA" OR "16S rRNA gene" OR "16S rRNA sequencing" OR "Metagenome" OR "Metagenomics" OR "High-Throughput Nucleotide Sequencing" OR "next generation sequencing" OR "Oral Microbiota" OR "microbial interactions" OR "host microbial interactions" OR "Saliva / microbiology" OR "metabolomics" OR "metabolomic" OR "Host microbiota" OR "Host microbiome")	69
**Embase**	('adults'/exp OR 'adults' OR 'adult'/exp OR 'adult') AND ('saliva'/exp OR 'saliva' OR 'salivary' OR 'salivary samples' OR 'saliva sample') AND ('salivary glucose' OR 'diabetes type 2' OR 'diabetes mellitus' OR 't2dm' OR 'diabetes' OR 'hyperglycemia' OR 'hyperglycemic') AND ('microbiota' OR 'community, microbial' OR 'microbial community compositions' OR 'community structure, microbial' OR 'microbiome, human' OR 'microbiome' OR 'human microbiome' OR 'dna bacterial' OR 'salivary microbiota' OR 'salivary microbiome' OR '16s' OR '16s rrna' OR '16s rrna gene' OR '16s rrna sequencing' OR 'metagenome' OR 'metagenomics' OR 'high-throughput nucleotide sequencing' OR 'next generation sequencing' OR 'oral microbiota' OR 'microbial interactions' OR 'host microbial interactions' OR 'saliva / microbiology' OR 'metabolomics' OR 'metabolomic' OR 'host microbiota' OR 'host microbiome')	109
**Scopus**	("microbiota" OR "community, microbial" OR "microbial community compositions" OR "community structure, microbial" OR "microbiome, human" OR "microbiome" OR "human microbiome" OR "dna bacterial" OR "salivary microbiota" OR "salivary microbiome" OR "16s" OR "16s rrna" OR "16s rrna gene" OR "16s rrna sequencing" OR "metagenome" OR "metagenomics" OR "high-throughput nucleotide sequencing" OR "next generation sequencing" OR "oral microbiota" OR "microbial interactions" OR "host microbial interactions" OR "saliva / microbiology" OR "metabolomics" OR "metabolomic" OR "host microbiota" OR "host microbiome") AND ("salivary glucose" OR "diabetes type 2" OR "diabetes mellitus" OR "t2dm" OR "diabetes" OR "hyperglycemia" OR "hyperglycemic") AND ("adults" OR "adult") AND ("saliva" OR "salivary" OR "salivary samples" OR "saliva sample")	80
**LILACS**	("microbiota" OR "microbiome") AND ("diabetes type 2" OR "diabetes tipo 2" OR "diabetes mellitus" OR "t2dm" OR "diabetes" OR "hyperglycemia" OR "hiperglicemia OR "hyperglycemic") AND ("saliva" OR "salivary")	14
**Livivo**	"Saliva" AND "Diabetes Type 2" OR "diabetes mellitus" AND "Oral Microbiota"	408

**Google Scholar**	saliva OR salivary AND diabetes mellitus AND microbiota	100
**ProQuest**	“Saliva” AND “Diabetes Type 2” AND “Oral Microbiota”	28

Supplementary Table S2. Excluded studies and reason for exclusion (n= 47).

**Author, year**	**Reason for exclusion**
Aitken-Saavedra et al. 2018 [46]	1
Almusawi et al .2018 [47]	1
Almusawi et al. 2020 [48]	1
Amato et al. 1983 [49]	3
Baena-Monroy et al. 2005 [50]	4
Baig et al. 2011 [51]	1
Balan et al. 2015 [52]	1
Barnes et al. 2014 [53]	5
Bernabe et al. 2019 [54]	6
Bianchi et al. 2016 [55]	1
Bremenkamp et al. 2011 [56]	1
Chouhan et al. 2019 [57]	1
Crusell et al. 2020 [58]	7
Darwazeh et al. 1991 [59]	1
Davila-Perez et al. 2007 [60]	2
Farizal et al. 2017 [61]	1
Goodson et al. 2017 [62]	7
Heggendorn et al. 2013 [63]	7
Hintao et al. 2007 [64]	1
Janem et al. 2017 [65]	8
Javed et al. 2009 [66]	2
Kampoo et al. 2014 [67]	1
Khovidhunkit et al. 2009 [68]	1
Latti et al. 2018 [69]	1
Liao et al. 2013 [70]	9
Lira-Junior et al. 2018 [71]	7
Minty et al. 2019 [72]	7
Mizushiri et al. 2019 [73]	6

Nabee et al. 2017 [74]	1
Naik et al. 2014 [75]	1
Omori et al. 2021 [76]	2
Popova et al. 2020 [77]	6
Radhakrishnan et al. 2019 [78]	6
Rajakumari et al. 2016 [79]	2
Salminen et al. 2014 [80]	7
Salminen et al. 2015 [81]	7
Samniengn et al. 2017 [82]	1
Shanker et al. 2013 [83]	10
Shenoy et al. 2014 [84]	1
Soni et al. 2019 [85]	6
Suarez et al 2013 [86]	1
Suárez et al. 2013 [87]	1
Tatarakis et al. 2014 [88]	2
Vijayalakshmi et al. 2020 [89]	1
Wang et al. 2018 [90]	7
Wang et al. 2019 [91]	7
Wei et al. 2020 [92]	1

Reason for exclusion legend:

1- Studies not using molecular methods for microbiome analysis and/or studies using culture as a method to evaluate the salivary microbiome (n = 22)
2- Samples other than saliva (biofilm, crevicular fluid, etc.), or different collection method (n = 5)
3- Complete paper not found (n = 1)
4- Include samples from individuals with prosthesis (n = 1)
5- Metabolomics data (n = 1)
6- Abstract, poster, banner or article published in event's annals (n = 5)
7- Studies of individuals with type 1 or gestational DM, or not clear if the diabetes group includes individuals other than T2D (n = 7)
8- Population including children/adolescents (n = 1)
9- Article published in non-latin alphabet (n = 1)
10- Does not show data of acid-associated microbiota (n = 1)

Supplementary Table S3. Quality assessment of the individual included studies (n = 12) using The Joanna Briggs Institute Critical Appraisal Checklist for Cross-sectional Studies.

	Al-Rawi l, 2017	Sun, 2020	Anbalagan, 2017	Yang, 2020	Kori, 2020	Chumponsuk [illegible]	Almeida- [illegible]	Liu, 2021	Ogawa, 2017	Sabharwal, 2019	Saeb, 2019	Vieira Lima, [illegible]
**Q1 -** Were the criteria for inclusion in the sample clearly defined? *	Y	Y	N	Y	N	Y	U	Y	N	Y	Y	Y
**Q2 -** Were the study subjects and the setting described in detail? *	N	Y	N	Y	N	Y	N	U	N	N	Y	Y
**Q3 -** Was the exposure measured in a valid and reliable way? *	N	Y	Y	Y	Y	Y	Y	Y	Y	U	Y	Y
**Q4 -** Were objective, standard criteria used for measurement of the condition? *	N	Y	Y	Y	U	Y	Y	Y	U	N	Y	Y
**Q5 -** Were confounding factors identified?	N	N	Y	N	N	U	N	U	N	N	Y	Y
**Q6 -** Were strategies to deal with confounding factors stated?	N	N	Y	N	N	N	N	U	N	N	Y	Y
**Q7 -** Were the outcomes measured in a valid and reliable way?	Y	Y	Y	Y	Y	Y	Y	Y	Y	Y	Y	Y
**Q8 -** Was appropriate statistical analysis used?	Y	Y	Y	Y	Y	Y	Y	Y	Y	Y	Y	Y
**Methodological quality**	**L**	**H**	**L**	**M**	**L**	**M**	**L**	**M**	**L**	**L**	**H**	**H**

Critical domains (*); yes (Y), no (N), unclear (U). Methodological quality was categorized as high (H), low (L) or moderate (M) according to critical domain of each question. Criteria adopted to this systematic review for considering a low methodological quality was two "no" in any domain, or one "no" and one "unclear" in critical domains. High methodological quality was considered when an article got a maximum one "no" answer or two "unclear" answers in non-critical domains. The other ones were classified as moderate.
